# Brainstem glucose metabolism predicts reward dependence scores in treatment-resistant major depression

**DOI:** 10.1017/S0033291720005425

**Published:** 2022-10

**Authors:** Guo-Rong Wu, Chris Baeken

**Affiliations:** 1 Faculty of Psychology, Key Laboratory of Cognition and Personality, Southwest University, Chongqing, China; 2 Department of Psychiatry University Hospital (UZBrussel), Brussels, Belgium; 3 Ghent Experimental Psychiatry (GHEP) Lab, Ghent, Belgium; 4 Department of Head and Skin, Ghent University Hospital, Ghent University, Ghent, Belgium; 5 Department of Electrical Engineering, Eindhoven University of Technology, Eindhoven, The Netherlands

**Keywords:** ^18^FDG PET, depression, reward dependence, TCI, treatment resistance

## Abstract

**Background:**

It has been suggested that individual differences in temperament could be involved in the (non-)response to antidepressant (AD) treatment. However, how neurobiological processes such as brain glucose metabolism may relate to personality features in the treatment-resistant depressed (TRD) state remains largely unclear.

**Methods:**

To examine how brainstem metabolism in the TRD state may predict Cloninger's temperament dimensions Harm Avoidance (HA), Novelty Seeking (NS), and Reward Dependence (RD), we collected ^18^fluorodeoxyglucose positron emission tomography (^18^FDG PET) scans in 40 AD-free TRD patients. All participants were assessed with the Temperament and Character Inventory (TCI). We applied a multiple kernel learning (MKL) regression to predict the HA, NS, and RD from brainstem metabolic activity, the origin of respectively serotonergic, dopaminergic, and noradrenergic neurotransmitter (NT) systems.

**Results:**

The MKL model was able to significantly predict RD but not HA and NS from the brainstem metabolic activity. The MKL pattern regression model identified increased metabolic activity in the pontine nuclei and locus coeruleus, the medial reticular formation, the dorsal/median raphe, and the ventral tegmental area that contributed to the predictions of RD.

**Conclusions:**

The MKL algorithm identified a likely metabolic marker in the brainstem for RD in major depression. Although ^18^FDG PET does not investigate specific NT systems, the predictive value of brainstem glucose metabolism on RD scores however indicates that this temperament dimension in the TRD state could be mediated by different monoaminergic systems, all involved in higher order reward-related behavior.

## Introduction

Major depressive disorder (MDD) is a severe mental health problem affecting millions worldwide. Despite a variety of treatment modalities, depression is prone to become a difficult-to-treat and chronic psychiatric illness, often referred to as treatment-resistant depression (TRD) (Akil et al., 2018). Although still an issue of debate, personality features may not only add to the vulnerability to develop depression (Newton-Howes, Tyrer, & Johnson, 2006), it may drive treatment outcome and clinical non-response (Cloninger, Svrakic, & Przybeck, 2006). Depressed patients with a comorbid personality disorder may respond less well to treatment, regardless of the chosen intervention (Rosenbluth, Macqueen, McIntyre, Beaulieu, & Schaffer, 2012). Furthermore, a long history of depression, with multiple episodes of resistance to treatment may induce personality changes within patients, which can be stable over time (Teraishi et al., 2015; Zaninotto et al., 2016), and this might also influence the results concerning the role of personality traits in relation to clinical outcome.

In pharmacotherapy research, personality dimensions have been evaluated to examine their ability to guide or even predict clinical outcome in MDD (Kaneda, Yasui-Furukori, Nakagami, Sato, & Kaneko, 2011; Mulder, 2002; Mulder, Joyce, & Luty, 2003). For instance, concerning the Temperament and Character Inventory (TCI; Cloninger, Przybeck, Svrakic, & Wetzel, 1994), higher scores on the temperament dimension Harm Avoidance (HA) has been related to an increased occurrence of mood and anxiety disorders (Hansenne et al., 1999; Hirano et al., 2002; Nery et al., 2009; Spittlehouse et al., 2010). HA refers to the inhibition and cessation of behavior and is theoretically associated with the serotonergic system. Depressed patients displaying higher scores on Novelty Seeking (NS) – associated with the dopaminergic system and implying the activation and initiation of behavior – have been, however not consistently, linked with a more favorable antidepressant (AD) treatment outcome (Mulder et al., 2003). Lower scores on the temperament dimension Reward Dependence (RD) – thought to be related to noradrenergic functioning and described as the maintenance and continuation of ongoing behavior – could be more at risk for TRD (Takahashi et al., 2013). The temperament dimension Persistence, formerly part of the RD personality construct but is currently regarded as a separate temperament dimension, has not been related to any monoaminergic neurotransmitter (NT) system yet (Cloninger et al., 1994). Importantly, the brainstem is home to a group of modulatory NT pathways, such as those arising from the raphe nuclei (serotonergic), ventral tegmental area (VTA) (dopaminergic), and locus coeruleus (LC) (noradrenergic), which form a modulatory network that coordinates interactions between other networks in the brain via the hypothalamus and thalamus providing further input in the frontal, cingulate, and other regions of the cerebral cortex (Venkatraman, Edlow, & Immordino-Yang, 2017).

In a recent large cohort study conducted by Balestri et al. (2019), these authors showed that MDD patients who did not remit after adequate trials of AD continued to display high scores on HA. They also found that clinical non-response was associated with high scores on HA and low scores on RD, but most importantly they showed that lower scores on RD were found to be related to TRD. Unfortunately, no neurobiological markers were included. ^18^Fluorodeoxyglucose positron emission tomography (^18^FDG PET) is a well-known neuroimaging method which has been shown to be very insightful to detect brain metabolic disturbances in the depressed state (Mayberg, 2009; Sublette et al., 2013). Several ^18^FDG PET studies have examined the relationship between the TCI and regional brain glucose metabolism in healthy subjects (Hakamata et al., 2009; Park, Park, & Kim, 2016; Youn et al., 2002). However, these studies employed univariate analytical techniques to identify brain–personality relationships that may mask within-group heterogeneity. Critically important for translational neuroscience, machine learning-based approaches may be better placed to predict personality traits using neuroimaging signatures at the individual subject level (Jiang et al., 2018).

Because it is largely unclear how regional glucose metabolism (CMRglc) may be implicated in the treatment-resistant depressed state and no studies have examined yet its influence on the prediction of temperament dimensions, we collected a group of well-defined TRD patients and we expected that metabolic activity in particular parts of the brain stem (and their respective NT systems) would significantly modulate the different temperament scores in TRD. We aimed to find CMRglc-based biomarkers for performing personalized prediction of temperament in TRD.

## Methods

### Participants

The study was approved by the ethics committee (UZBrussel) and all participants gave written informed consent. Forty AD-free depressed patients were included by using the Dutch version of the Mini-International Neuropsychiatric Interview (MINI; Sheehan et al., 1998) excluding bipolar and psychotic depression. Subjects with substance abuse/dependence were also not included. To evaluate depression severity, patients were assessed with the 17-item Hamilton Depression Rating Scale (HDRS; Hamilton, 1967). As described by Rush, Thase, & Dube (2003), all included MDD patients were considered at least stage III treatment-resistant: they had had a minimum of two unsuccessful treatment trials with a serotonin reuptake inhibitor/noradrenaline and/or serotonin reuptake inhibitor and one failed clinical trial with a tricyclic antidepressant. To avoid influences of concomitant AD medication, all patients went through a medication washout and they were AD-free for at least 2 weeks before the ^18^FDG PET scan. Right handedness was assessed with the Van Strien questionnaire (Van Strien & Van Beek, 2000). This study was part of a larger project investigating several neurobiological and neurocognitive markers in TRD.

### Assessment

Before performing the ^18^FDG PET scan, all participants were assessed with the HDRS (Hamilton, 1967) and with the Dutch version of the TCI (de la Rie, Duijsens, & Cloninger, 1998). The TCI contains three character dimensions: Cooperativeness, Self-Directedness, and Self-Transcendence, which are the complex result of learning processes, emotional maturation during one's entire life and comprise socio-cultural factors, and four temperament dimensions – which are thought to be largely hereditary, manifesting early in life and remaining largely stable throughout a lifetime – barely influenced by external factors (Cloninger, 1987; Cloninger, Svrakic, & Przybeck, 1993). Because only HA, NS, and RD are thought to be related to specific neurobiological (NTs) systems (Cloninger et al., 1994), we specifically focused on these three temperament dimensions. Given that gender differences in MDD are well documented and a proximally 17.1% of women and 9.4% of men worldwide will experience a depressive episode during their lifetime (Alonso, Lepine, & Committee, 2007), and given that differences have been reported on temperament dimensions, in the depressed as well as in the non-depressed state (Miettunen, Veijola, Lauronen, Kantojarvi, & Joukamaa, 2007), we incorporated gender (but also age) in all our analyses.

All behavioral data were analyzed with SPSS 25 (Statistical Package for the Social Sciences, IBM, Chicago, USA). The significance level was set at *p* ⩽ 0.05, two-tailed, for all analyses (see Table 1).

**Table 1.** Demographics

	TRD patients	Females	Males	*p* value
Gender	39	26	13	–
Age	47.77 (10.37)	48.65 (13.40)	45.62 (12.50)	0.37
HDRS	25.13 (5.14)	26.15 (9.89)	23.08 (5.07)	0.08
Duration depressive episode (years)	6.51 (6.61)	5.79 (4.26)	7.96 (8.97)	0.46
NS^a^	15.97 (5.80)	17.08 (5.76)	13.77 (5.43)	0.09
HA^a^	26.59 (8.00)	27.81 (7.66)	24.15 (8.39)	0.18
RD^a^	15.92 (3.27)	15.92 (3.17)	15.92 (3.60)	0.99
P	4.77 (2.51)	4.81 (2.53)	4.69 (2.56)	0.89
SD	22.62 (7.12)	21.69 (6.92)	24.46 (7.43)	0.26
CO	30.51 (4.87)	29.73 (5.14)	32.08 (3.99)	0.16
ST	11.64 (6.34)	12.46 (6.32)	10.00 (6.30)	0.26

MDD, major depressive disorder; HDRS, Hamilton Depression Rating Scale; NS, novelty seeking; HA, harm avoidance; RD, reward dependence; P, persistence; SD, self-directedness; CO, cooperativeness; ST, self-transcendence.

Ratio's, means and standard deviations are provided.

a Temperament dimensions focused on for this study.

### ^18^FDG-PET brain imaging

Aligned with our previous CMRglc research (Baeken et al., 2009, 2015; Baeken, Wu, & De Raedt, 2018; van Heeringen, Wu, Vervaet, Vanderhasselt, & Baeken, 2017), static ^18^FDG-PET scan imaging was performed by a Siemens Ecat Accel scanner 30 min after intravenous tracer administration (222 MBq ^18^FDG). All ^18^FDG-PET scans were performed between 9:00 and 11:00 h and all participants had to lie supine with their eyes closed. Timings of producing ^18^FDG, injecting, and scanning were in accordance with the European Association of Nuclear Medicine Neuroimaging guidelines (Varrone et al., 2009). Emission data were obtained in 3D mode over 10 min. For transmission, germanium-68 sources (3 × 185 MBq; decay corrected) were used and data were acquired in 2D mode over 3 min. The emission data were reconstructed iteratively (OSEM 10 iterations, 32 subsets) applying a post-reconstruction filter (Gaussian filter 6 mm full-width-at-half-maximum). For the transmission, we used filtered back reconstruction and all data were subsequently segmented into regions with similar attenuation factors. To obtain attenuation correction factors for each line of response, these segmented images were forward projected. The pixel size was 2.57 mm transaxial and 3.38 mm axial. Each scan was projected onto a normalized brain template with the SPM12 (Statistical Parametric Mapping, Welcome Department of Imaging Neuroscience, University College of London, UK) software. The voxel-wise metabolic activity in the brain stem was extracted for individual prediction using the Desikan–Killiany Atlas (Desikan et al., 2006).

### Individualized prediction

We employed a sparse multiple kernel learning (MKL) with elastic-net regularization to select the minimal set of voxels that best predicted temperament scores, implementing in the SpicyMKL toolbox (version 3; Suzuki & Tomioka, 2011). A nested cross-validation (CV) was used to perform hyperparameter optimization (10-fold CV for the inner loop, leave-one-out for the outer loop). Age, gender, and mean global CMRglc value were combined in one single linear kernel. For each voxel in the brainstem, a linear kernel was computed. A voxel with higher kernel weight (unsigned) can be interpretative as a higher contribution to the predictive model. The Pearson correlation coefficient (*r*) and root mean squared error (rMSE) were calculated between the actual and the predicted temperament scores for the overall predictive performance. Permutation tests were carried out to assess the statistical significance of the correlation coefficient and rMSE (randomly shuffle temperament scores 1000 times).

## Results

For one 56-year-old male patient, the ^18^FDG-PET scan showed severe artifacts and was not suitable for analysis. Consequently, this patient was removed from the analysis.

### ^18^FDG-PET brain imaging results

**Fig. 1.**
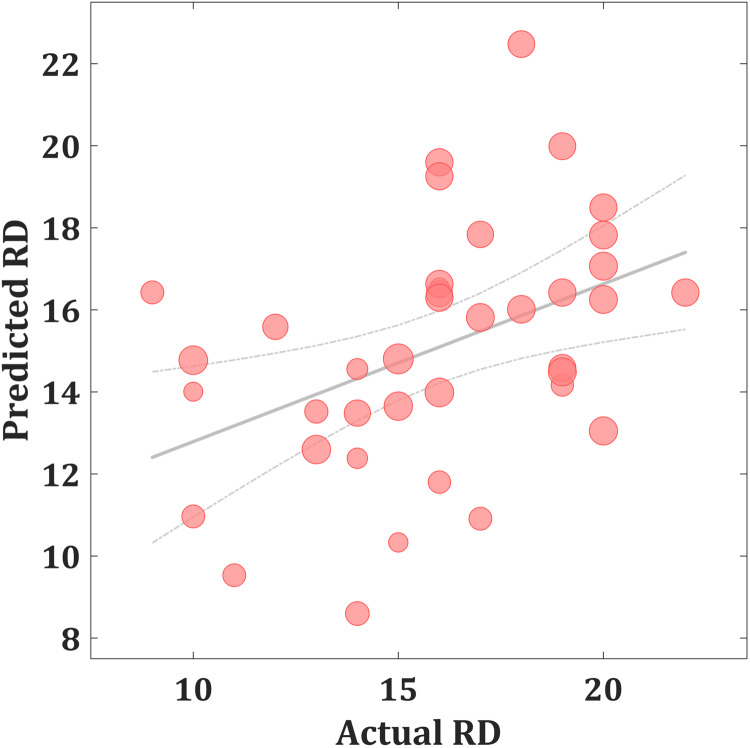
Scatter plot showing actual and predicted reward dependence (RD) scores (*r* = 0.425, *p* = 0.004; *r*MSE = 3.418, *p* = 0.015). The size of scatter point is proportional to the mean global CMRglc value.

**Fig. 2.**
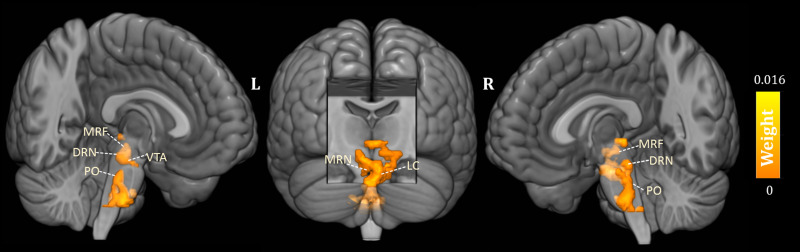
Three-dimensional overview of voxel-wise kernel weight in the brainstem for reward dependence (RD) prediction, with cluster size >10 voxels. The color represents the relative informativeness of different voxels. PO, pontine nuclei; LC, locus coeruleus; MRF, medial reticular formation; DRN, dorsal raphe nuclei; MRN, median raphe nuclei; VTA, the ventral tegmental area.

As shown in Fig. 1, the predicted RD scores significantly correlated with the actual RD scores (*r* = 0.425, *p* = 0.004; *r*MSE = 3.418, *p* = 0.015). Contributing to the predictions of RD, the MKL regression identified metabolic activity in the pontine nuclei (PO) and LC, the medial reticular formation (MRF), the dorsal/median raphe nuclei, the VTA, red nucleus, and substantia nigra [Fig. 2, only those voxels that survived at CV procedure are reported, the spatial location is identified by AAL3 (Rolls, Huang, Lin, Feng, & Joliot, 2020) and the Harvard Ascending Arousal Network Atlas (Edlow et al., 2012)]. In addition, the age, gender, and mean global CMRglc value also contribute to MKL prediction (4.64% of total weight). The voxel-wise correlations indicated that the metabolic activity in these regions was positively correlated with RD scores. In addition, brain stem CMRglc was not predictive for HA (*r* = 0.164, *p* = 0.115; *r*MSE = 8.34, *p* = 0.152) and NS (*r* = −0.122, *p* = 0.516; *r*MSE = 6.26, *p* = 0.533) scores.

## Discussion

This is the first study applying the MKL algorithm to predict the individual differences in temperament scores based on brainstem metabolic activity in TRD. The MKL model was able to predict the individual scores on RD from the brainstem metabolic activity, but brainstem CMRglc was not predictive for the individual scores on HA and NS. More precisely, we found that individual differences in RD are closely linked to metabolic increases in the raphe nuclei, the PO/LC, and the VTA, respectively, suggesting the implication of the serotonergic, noradrenergic, and dopaminergic system. These findings provide additional support that, at least in our case in the treatment-resistant depressed state, this temperament dimension shows significant increased CMRglc in brainstem regions associated with different monoaminergic NT systems (Samochowiec et al., 2001). Indeed, research not always has found the association between temperaments and their respective theoretically NT systems, and it remains an open question whether specific individual traits can be attributed to only one monoaminergic system (Robbins, 2018).

According to Cloningers' theory of personality, the temperament dimension RD has been linked with noradrenergic activity (Cloninger et al., 1994). Norepinephrine, in addition to its effects on sensory processing and waking behavior, it contributes to cognition, flexibility, and mnemonic processes, and dense noradrenergic innervations in the prefrontal cortical and hippocampal areas are thought to modulate these functions (Borodovitsyna, Flamini, & Chandler, 2017). The personality temperament RD has been related to the formation of conditioned signals of reward (Gerra et al., 2000) and may be implicated to maintain the behavior previously associated with reward (Bond, 2001). Subjects with lower scores on RD are described as less sentimental and less socially attached; they generally are less dependent on the approval of others, and those with higher scores than average are clinically characterized as eager to help and please others (Hansenne et al., 1999). Given that RD also reflects the maintenance of socially rewarded behavior, our ^18^FDG-PET findings in the LC and the pontine noradrenergic nuclei suggests that in the treatment-resistant state, this could be mediated by the noradrenergic system, reflecting behavioral flexibility in social contacts. Besides that pontine microbleeds in stroke may lead to depression (Tang et al., 2013, 2014), the PO are the largest of the precerebellar nuclei, providing the principal input to the cerebellum (Kratochwil, Maheshwari, & Rijli, 2017; Schwarz & Thier, 1999). Not surprisingly that current evidence also points to an important role of the cerebellum in the processing of social behaviors (Adamaszek et al., 2017).

It is of interest to note that metabolic activity in serotonergic and dopaminergic-related brainstem structures was also predictive for RD scores when clinically depressed. Serotonin is considered as a fundamental mediator of emotional, motivational, and cognitive aspects of reward representation (Kranz, Kasper, & Lanzenberger, 2010). The increased CMRglc in the raphe nuclei is suggestive for the involvement of the serotonergic system in this temperament construct in the desire for social relationships (Gerretsen et al., 2010). It encodes reward probability, reward value and cost, and how beneficial the current environmental context may be (Luo, Li, & Zhong, 2016). Besides that the dorsal raphe nucleus (DRN), the largest serotonergic nucleus in the brainstem, strongly moderates reinforcement learning, its neuronal activity has been associated with reward size (Nagai et al., 2020) and reward waiting (Luo, Zhou, & Liu, 2015). Together with the medial raphe nuclei (MRN), the DRN modulates dopaminergic activity in the VTA, a region which has been primarily implicated in reward-motivated behavior, similar to the red nucleus and the substantia nigra (Browne et al., 2019; Cha et al., 2014). Of interest, it is the interaction between the serotonergic and the dopaminergic system that controls the behavioral response to reward (or punishment) (Macoveanu, 2014), potentially mediated by excitatory glutamatergic neurons (Wang et al., 2019).

Although we only have focused on brainstem CMRglc, the positive relationship between RD scores and several specific brainstem areas associated with specific monoaminergic NT involvement suggest that, at least in the TRD state, the temperament RD construct goes beyond the noradrenergic system. This assumption is partly supported by the predictive metabolic activity of RD in the MRF, a medial brainstem network part of the emotional motor system mediated by multiple NT systems, also moderating emotional arousal and maintenance of consciousness (Venkatraman et al., 2017). Because we only measured CMRglc and no specific monoaminergic system, we can only assume that the higher metabolic activity predictive for higher scores on RD may represent higher order reward-related behavior and is mediated by noradrenaline, serotonin, and dopamine.

Our study had some major advantages: all TRD patients were AD-free on the time of scanning, and we implemented an MKL approach to identify CMRglc in brainstem regions predictive to determine temperament at the individual level. This overcomes the limitations of using univariate analysis which possesses limited sensitivity to identify subtle and spatially distributed effects, which could be important in complex personality dimensions with complex behavioral outcomes, involving multiple brain stem areas with a variety of functions (Fernandes et al., 2017; Jiang et al., 2018). MKL avoids the traditional in-sample correlation approach, prone to overfitting and not always allowing for out-of-sample generalizations, and not readily applicable to use on the individualized level. Notwithstanding, our CMRglc results cannot be generalized to the non-depressed state as it has been reported that RD scores may differ between resistant and non-resistant depressed samples (Balestri et al., 2019). Of course, because we used ^18^FDG PET as a tracer and no specific ligand to quantify any of the potentially involved NT systems, our brainstem CMRglc findings cannot specifically be attributed to NT processes. Given the *a priori* hypothesis of brainstem involvement, we cannot make claims on CMRglc predictions related to temperaments in other parts of the brain. Also, given the relatively poor resolution of ^18^FDG PET and that each scan was projected onto a normalized brain template and not to the individual anatomical scan, all result interpretations should be done cautiously. Lastly, the TCI was merely used as a screening for personality temperaments and it was not intended for an actual diagnosis of an underlying personality disorder.

In conclusion, our MKL model showed that brainstem CMRglc was predictive only for the temperament dimension RD. The predictive value of brainstem metabolic activity on RD scores indicates that this temperament dimension could be mediated by different monoaminergic systems. Our findings expand our neurobiological insight that in the treatment-resistant depressed state, potentially other NT systems besides the noradrenergic system may contribute to with the expressed desire for social relationships (Gerretsen et al., 2010). Machine learning paradigms may have good potential to further elaborate on the neurobiological mechanisms of personality. Future brain imaging studies using specific NT ligands are needed not only to investigate whether our findings on temperament also hold in the healthy and non-treatment-resistant depressed state.
